# Formation of Annual Ring Eccentricity in Coarse Roots within the Root Cage of *Pinus ponderosa* Growing on Slopes

**DOI:** 10.3390/plants9020181

**Published:** 2020-02-02

**Authors:** Antonio Montagnoli, Bruno Lasserre, Gabriella Sferra, Donato Chiatante, Gabriella Stefania Scippa, Mattia Terzaghi, R. Kasten Dumroese

**Affiliations:** 1 Department of Biotechnology and Life Science, University of Insubria, Via Dunant, 3–21100 Varese, Italy; donato.chiatante@uninsubria.it; 2 Department of Biosciences and Territory, University of Molise, Contrada Fonte Lappone, 86090 Pesche, Isernia, Italy; lasserre@unimol.it (B.L.); gabriella.sferra@unimol.it (G.S.); scippa@unimol.it (G.S.S.); 3 Department of Chemistry and Biology ‘A. Zambelli’, University of Salerno, 84084 Fisciano, Salerno, Italy; mterzaghi@unisa.it; 4 Rocky Mountain Research Station, U.S. Department of Agriculture Forest Service, 1221 South Main Street, Moscow, ID 83843, USA; kas.dumroese@usda.gov

**Keywords:** root system, *Pinus ponderosa*, reaction wood, root architecture, annual rings, mechanical forces

## Abstract

The coarse roots of *Pinus ponderosa* included in the cage are the ones most involved in tree stability. This study explored the variations in traits, such as volume, cross-sectional area, and radius length of cage roots, and used those data to develop a mathematical model to better understand the type of forces occurring for each shallow lateral root segment belonging to different quadrants of the three-dimensional (3D) root system architecture. The pattern and intensity of these forces were modelled along the root segment from the branching point to the cage edge. Data of root cage volume in the upper 30 cm of soil showed a higher value in the downslope and windward quadrant while, at a deeper soil depth (>30 cm), we found higher values in both upslope and leeward quadrants. The analysis of radius length and the cross-sectional area of the shallow lateral roots revealed the presence of a considerable degree of eccentricity of the annual rings at the branching point and at the cage edge. This eccentricity is due to the formation of compression wood, and the eccentricity changes from the top portion at the branching point to the bottom portion at the cage edge, which we hypothesize may be a response to the variation in mechanical forces occurring in the various zones of the cage. This hypothesis is supported by a mathematical model that shows how the pattern and intensity of different types of mechanical forces are present within the various quadrants of the same root system from the taproot to the cage edge.

## 1. Introduction

Global changes have increased the number of storms that annually affect forest ecosystems [1,2], inducing tree uprooting and losses of timber to an alarming level [3,4]. As a consequence, greater attention to tree anchorage has entered breeding and nurseries programs [5,6] and silvicultural practices [7,8,9]. While the need to stress the role played by roots not only in tree anchorage but also in nutrition and reserve storage [10,11,12] is clear, the number of studies regarding coarse roots is still too small, and even important aspects of their development remain largely unknown.

The lack of root studies is likely a combination of the hostile medium (i.e., the soil) where roots live, that hampers “in situ” investigations, and the limited commercial value of root wood. Nevertheless, sufficient evidence demonstrates that the three-dimensional (3D) architecture of a root system undergoes variation in response to the plant’s demand for water and nutrients [13]. Moreover, it is becoming equally clear that 3D root architecture is strongly affected by several other environmental factors, such as the mechanical stresses deriving from the weight of above-ground organs or from the wind force acting on the tree canopy. In the case of mechanical stresses, the role of roots is to transfer these forces into the soil rapidly (and efficiently) to avoid uprooting. Within a root system, the 3D architecture represents all the available and possible routes (these being the root axes) [14] followed by the forces to be dispersed into the soil [15,16,17,18]. Concurrent with the mechanical stress, other stressors, such as drought or soil’s chemical–physical properties, can affect root system 3D architecture [19,20,21,22].

Root systems have been categorized into three different groups based on their 3D architecture shape: heart, plate, and taproot [23]. These categories are still commonly used despite acknowledgement that, in consequence of the above-mentioned plasticity, the inclusion of a root system in a specific category represents only the final result of an adaptation to all factors that have affected the roots [10]. In fact, we recently demonstrated that *Pinus ponderosa* trees growing in the same stand used for this study (see Materials and Methods) can deploy three different types of root architecture, which could be considered as subclasses of a single taproot category as classified by [18]. In this study [10], we suggested that root 3D architecture could represent different responses to mechanical forces due to slope and wind, similar to responses observed in the 3D architecture of *Quercus pubescens* [23]; however, a review of all possible factors able to induce modification of 3D root architecture is well beyond the scope of this paper.

Computer modelling in the last two decades has highlighted the importance of understanding the mechanical contribution to anchorage provided by each type of component present in the root system: taproot, shallow lateral root, oblique lateral root, and sinker lateral root [24,25,26]. In this sense, the work of Danjon et al. [27] with *Pinus pinaster* that suggests that the most important contributions to tree anchorage are provided by all the roots included in a cylindrical zone surrounding the taproot, i.e., the “cage”, is particularly relevant. Therefore, the development of any provisional model must include the role played by roots within the cage, achieved by analyzing their traits [28,29,30,31].

A good approach toward understanding the effect of mechanical forces upon root development is to use current knowledge concerning the response of stem tissue to mechanical forces. A large body of literature reports the production of reaction wood in the stem of angiosperms (named tension wood) and gymnosperms (named compression wood) [32,33] in response to mechanical stresses. Reaction wood corresponds to an anomalous production of wood richer in mechanical fibers with respect to normal wood, and less cellulose and more lignin in the cell walls of vessels than those occurring in normal wood [34]. The function of reaction wood is to counteract mechanical stress on the stem (or the branches), and therefore it is reasonable to assume that mechanical forces could induce the same effect on root tissues, too. The scarcity of literature concerning the formation of reaction wood in roots is somewhat surprising, given that the presence of compression and tension wood in roots was reported more than a half-century ago [34]. These earlier studies were reviewed by Timell [35], who states that the reaction wood in roots of conifers is matched by a great eccentricity, with the radial growth occurring mainly on the upper side of the cross-section of root growing parallel to the soil. This wood asymmetry has been explained as the response to mechanical stress resulting from stem sway [36,37,38]. In the same review, Timell [35] states that annual ring eccentricity can change direction at a growing distance from the taproot branching point with a 260° variation in as little as 10 cm distance [39].

In addition, Nicoll and Dunn [40] show in *Picea sitchensis* that wind has a considerable effect on root system carbon allocation. Creber and Chaloner [41] suggest that the wood eccentricity observed in roots is always different from that observed in stems, as the amount of latewood found in roots is never much greater than the amount of early wood. However, the difficulties in accepting the occurrence of reaction wood in roots is due not only to the small number of publications available but also to the occurrence of a reduced amount of latewood with tracheids that tend to be round in their cross-section, unlike those observed in the stem [42]. In addition, the role of gravity in providing mechanical stress that induces the formation of compression wood in the stem overrides any other mechanical stimulus (including wind) [43]. 

The posture control of trees in response to mechanical forces that risk their stability is still poorly understood and requires an integrative interdisciplinary approach, as suggested by Moulia et al., [44,45]. Our study objective is to better understand how the root system develops and adapts to its rooting environment in order to achieve an efficient mechanical anchorage to the soil. Studies such as this are foundational to the development of a 3D root system model [26] where the mechanical stability of the trees can be studied, and ideally would enable land managers and researchers to foresee whether the anchorage of a specific tree at a particular time is sufficient to resist a worsening of environmental stressors. Our work presented here attempts to understand the role played by the root system in the anchorage of *P. ponderosa* trees growing on a slope in the presence of a constant seasonal wind. In particular, we examined the effect of mechanical stresses deriving from different sources, such as aboveground biomass, wind, and slope direction. We focused particularly on the role played by the roots present in the cage by measuring traits such as radius length, volume, and cross-sectional area. We also investigated the presence of eccentricity and difference in wood color in root cross-sections, which might be related to the formation of reaction wood. Finally, we developed a theoretical model to understand the type and distribution of mechanical forces acting on the root system architecture.

## 2. Materials and Methods

### 2.1. Site Description and Tree Sampling

We sampled eight, 32 year old *P. ponderosa* that were originally hand-planted on the University of Idaho Experimental Forest in northern Idaho, USA [lat 46.842240, long -116.871035; 1000 m elevation]. Dumroese et al. [10] and Montagnoli et al. [46] provide complete details of the plantation, soil, and site. Briefly, trees were growing on a 2 m × 2m spacing on 30–50% slopes on a northeast aspect. The soil was deep (~1.5 m to bedrock), having formed in volcanic ash above weathered granite. Annually, the mean temperature is 7.2 °C, the growing season is about 100 frost-free days, and precipitation is approximately 965 mL with a seasonal summer drought (July through September) [47]. West–southwest winds predominate during the growing season [48] (see 46 for a complete weather description). 

Trees were felled after inserting a screw into the stem at the ground line to record north. We used an air spade to excavate root systems to bedrock and to distances of approximately 1.5 m from the stump [10]. Roots still attached to soil were severed and the root systems were carefully moved to the U.S. Department of Agriculture, Rocky Mountain Research Station, Forestry Sciences Laboratory (Moscow, Idaho).

### 2.2. 3-Dimensional Analysis and Root Cage Definition

At the laboratory, we re-oriented root systems to their original position, and then digitized all roots with a proximal diameter ≥ 1 cm at the base using a low magnetic digitizer, transmitter, and receiver. Data were encoded in a standard format (MTG) commonly used to represent branching, topological (i.e., branching hierarchic structure) relationships [49]. Topology was coded using the “acropetal-development approach” [50] with the seed-origin radicle, the primary roots (-axis) or taproot designated order zero. Lateral roots emerging from the taproot were designated first-order roots, with second-order roots originating from these first-order laterals, and so on [51]. We analyzed the data using AMAPmod software, specially developed to handle plant architecture [52], and computed root cage volume from the 3D digitizing data of the entire root system.

**Figure 1:**
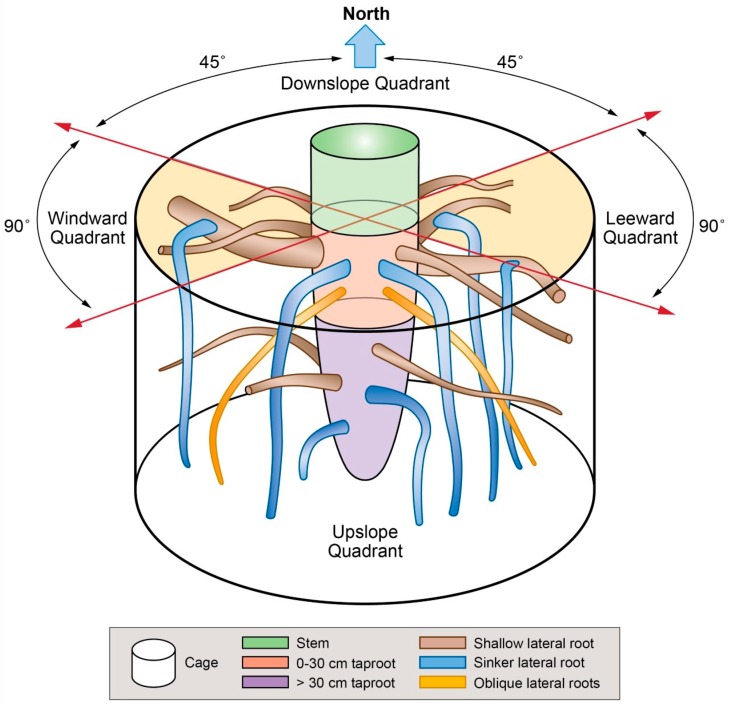
Schematic representation of the root cage and root types at two soil depths.

In this study, we focused on the root cage using a modified definition of cage, as defined by Danjon et al. [27]. In particular, the cage (Figure 1) includes all coarse roots (diameter > 1 cm) present in a cylindrical region centered at the taproot (or seminal root) (i.e., the largest vertical root connected directly to the stump). The cylinder had a diameter 2.2 times the value of the tree diameter breast height (DBH), as suggested by Danjon et al. [27], and a height that coincided with taproot length. The cylindrical region included the length of the shallow lateral roots, characterized by a rapid taper. The cage was divided by the crossing of two planes passing through the taproot axis, rotated 45° respect to the north (downslope) direction, and with northwest–southeast and northeast–southwest direction, resulting in dividing the space surrounding the taproot into four quadrants: downslope (with a north direction), upslope (south), windward (west), and leeward (east) (Figure 1). 

### 2.3. Root Volume, Radius Length, and Cross-Sectional Area (CSA) Measurements

Extracted data from AMAPmod software were exported to other software to perform specialized processing for volume estimation. Lateral roots were segregated according to their branching order (first-, second-, and third-order) and their point of origin (i.e., depth) along the taproot axis (<30 cm or >30 cm). Moreover, we made a distinction between the lateral roots branching in the four quadrants (downslope, upslope, windward, and leeward). Oriana software v. 4.02 (Kovach Computing Services; Kovach 1994) was used for the circular representation of root volume.

**Figure 2:**
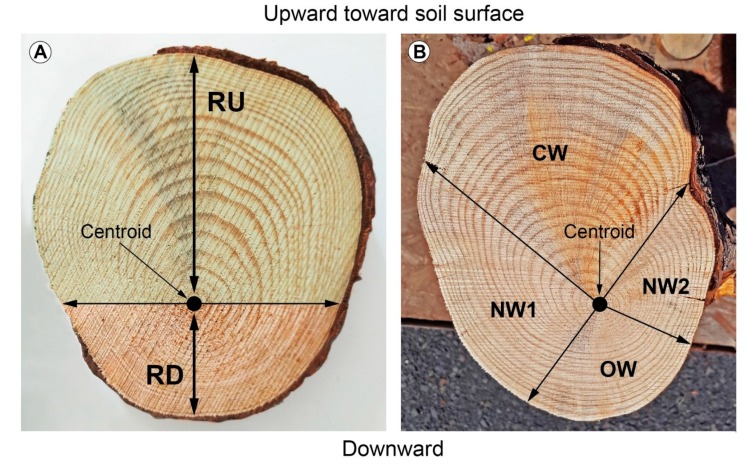
(**A**) Cross-sectional area was determined using software ImageJ. The first radius, RU, extended from the centroid of the section (the position of the oldest annual ring) upward toward the soil surface, whereas the second radius, RD, extended from the centroid downward, away from the soil surface. A transversal line through the centroid divided the cross-section into the top (light brown) and bottom (dark brown) portion of the wood section. (**B**) Compression wood (CW) occurred reverse of opposite wood (OW); these zones were separated by areas of normal wood (NW).

After being digitized, every root was detached from one side at its point of origin (named branching point) on the taproot and at the other side at a distance, coinciding with the end of the cage (named cage edge). The roots present in the cage were defined as shallow lateral roots when they elongated parallel to the ground, sinker lateral roots when they elongated vertically within the root cage, or oblique lateral roots when they elongated in the soil by moving away from the taproot [27] (Figure 1). The shallow lateral roots presented a rapid taper characterized by a slight curvature. Then, we measured the CSA of the roots growing within the cage from a photograph (Canon 1200D, Sigma 60Macro) after cutting a transverse section (2 cm thick) of the root cut at the branching point. Images were analyzed by Image J. On the same image, we investigated the presence of eccentricity of the annual rings by measuring the length of two radii. The first radius, RU, extended from the centroid of the section (the position of the oldest annual ring) upward toward the soil surface (Figure 2A). The second radius, RD, extended from the centroid downward away from the soil surface (Figure 2A). A transversal line through the centroid was set to divide the cross-section into two parts, and in this way it was possible to divide the overall CSA into two portions: top (toward the soil surface, light brown in Figure 2) and bottom (downward, dark brown in Figure 2A). 

The eccentricity of the wood due to the sum of the all annual rings was measured at both ends of the lateral root (branching point and cage edge) by using a digital caliper and measuring the distance from the centroid to the cork surface. In almost all of the shallow roots that branched from the taproot in the first 30 cm of soil depth, we notice that, in the zone presenting the maximum eccentricity, a slight difference in color was often visible. Although the investigation of reaction wood cannot be based solely on the presence of eccentricity and on a slight difference in wood color, these traits, together with anatomical structure, can be used to identify compression wood in conifers [35,53]. In this regard, we applied the method according to Timell [35], that consists of dividing the transverse section of wood into two portions, respectively indicated in Figure 2B as compression wood (CW) and opposite wood (OW), along with two portions of normal wood (NW1 and NW2). 

### 2.4. Modelling

**Figure 3:**
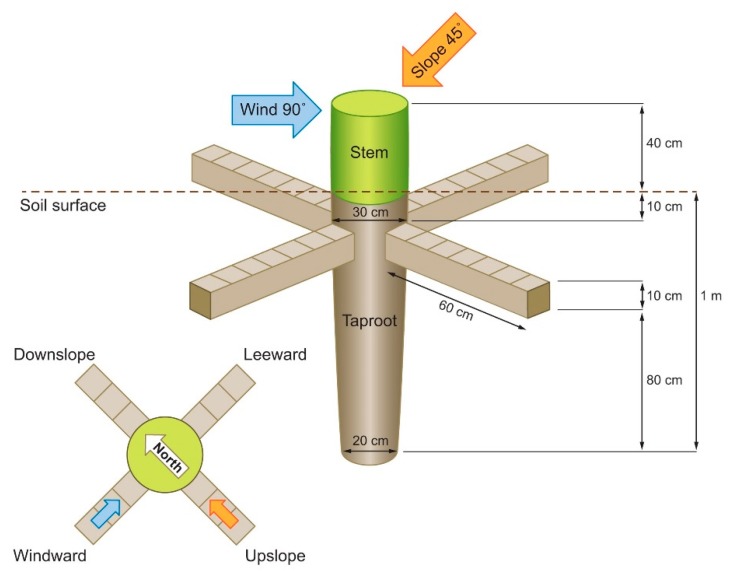
Model representing the distribution of mechanical forces affecting a root system formed by the taproot axis and four lateral roots branching from the taproot and elongating in the four cardinal directions.

Root modelling (Figure 3) was performed by means of Mecway software (version 11.0; Mecway Limited, New Zealand), a comprehensive user-friendly finite element analysis package [54]. A realistic 3D model of the root system was built by taking into account the diameters of sections across the taproot and lateral roots. The root diameters considered ranged from 30 cm (beginning of the taproot) to 20 cm (1 m deep in the ground). Four lateral roots with a diameter of 10 cm were included, one from each quadrant (i.e., upslope, downslope, windward, and leeward); their emission was set at 10 cm after the beginning of the taproot and a length of 60 cm from the emission point. Also, 40 cm of the shoot was included in the model.

Plant material was considered elastoplastic with a density of 1000 kg/m^3^, Young’s modulus of 5 GPa, and a Poisson ratio of 0.3, as previously reported [55]. Meshes constructed on plant diameter were used to construct a two-dimensional section that was revolved on its longitudinal axis in order to derive the 3D model. Lateral roots were obtained by extruding meshes on the surface of the taproot. Tree self-loading was applied uniformly on the top of the model. Tree loading related to the slope was included in the model as a pressure directed orthogonally at 45° in respect to the longitudinal axis of the shoot. The wind was included as a pressure orthogonal to the shoot, with a latitudinal angle of 45° with respect to the slope. Tension and compression forces were evaluated. 

### 2.5. Statistical Analysis

Mean and cumulative CSA and radius length data were neither normally distributed nor did they meet the assumption of homoscedasticity. Thus, data were square-root or log-transformed to ensure normal distributions and equal variances to allow the use of parametric statistics. A one-way ANOVA was used to compare root data obtained from different quadrants. Post hoc Least Significant Difference (LSD) tests were conducted to detect overall differences between upward and downward CSA values for all quadrants. Analyses were applied to a 95% significance level. Statistical analysis was carried out using a statistical software package SPSS 17.0 (SPSS Inc., Chicago, IL). Tension and compression forces were evaluated and plots and statistical analyses of stresses among upward and downward sides of each lateral root were derived by R environment. Stresses were compared by a *t*-student test (*p* < 0.05). 

## 3. Results

### 3.1. Radius Length, CSA, and Lateral Root Volume

During the removal of lateral roots from the taproot, we noticed the presence of eccentricity of the annual rings in the traces left by all the lateral roots along the taproot axis. This eccentricity was particularly evident when referring to the shallow lateral roots (i.e., lateral roots branching in the first 30 cm of taproot depth). Little or no eccentricity was found in sinker or oblique lateral roots.

**Figure 4:**
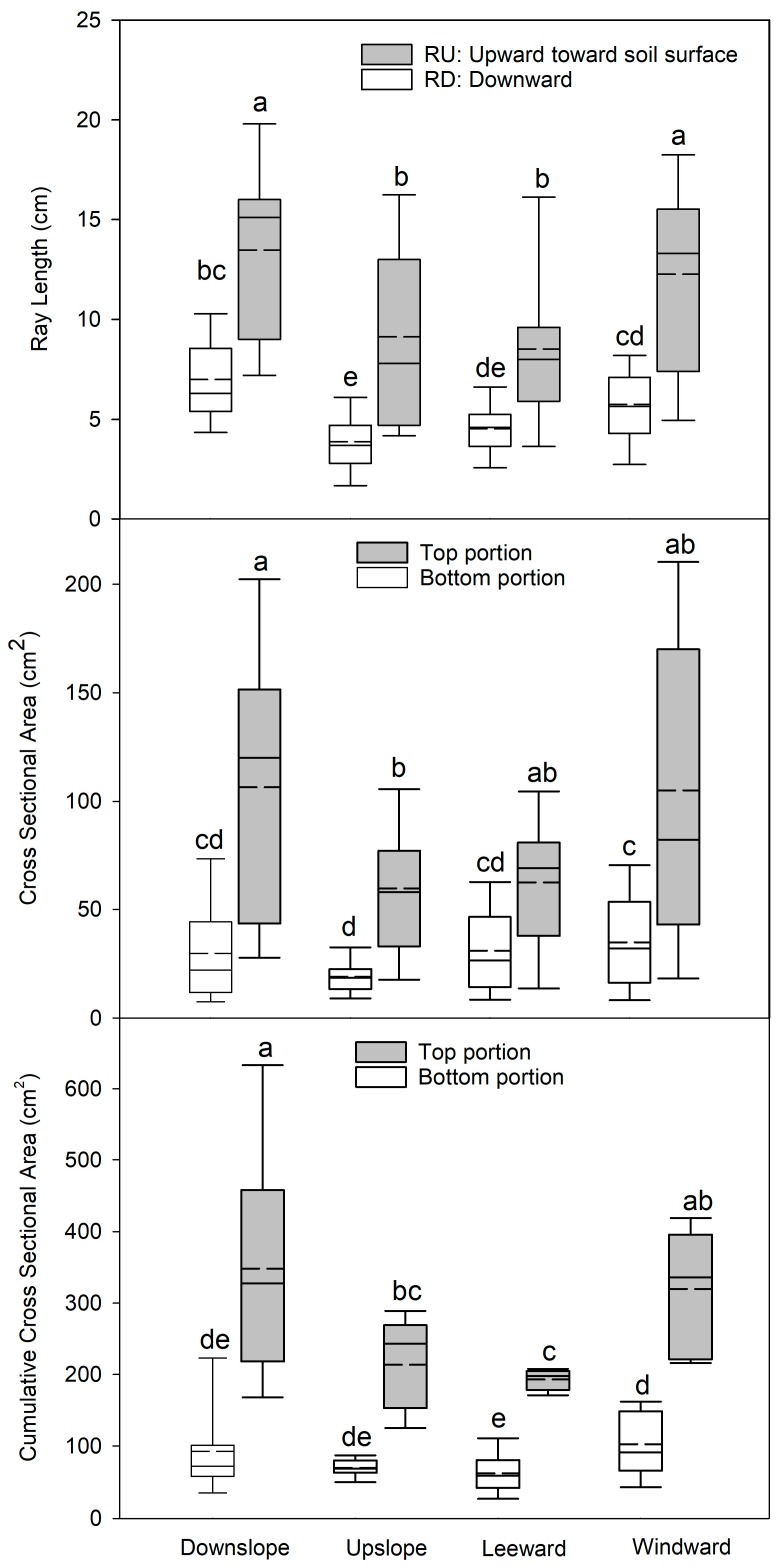
Length of RU (radius oriented upward toward the soil surface), RD (radius oriented downward away from the soil surface), and cross-sectional area (CSA) for the top and bottom portion of the traces left from the lateral roots on the taproot after their removal in the four cardinal quadrants of the 3D root architecture. Letters within each quadrant, and portion combination indicate significant differences (*p* < 0.05); vertical boxes represent approximately 50% of the observations and lines extending from each box are the upper and lower 25% of the distribution. Within each box, the solid horizontal line is the median value and the dotted line is the mean.

We found that all the traces presented an RU length significantly higher (*p* < 0.05) than RD in all the roots belonging to the four quadrants of the 3D root architecture (Figure 4). Moreover, the RU length was significantly higher (*p* < 0.05) in the roots belonging to the downslope and windward quadrants than those in the upslope and leeward quadrants. This was true also for the RD length, with the highest values measured for the roots belonging to the downslope and windward quadrants, although in the case of wind- and lee- ward quadrants this difference was not significant.

The analysis of the CSA of all traces left on the taproot by the lateral roots followed the same pattern observed with RU and RD length. In fact, the highest values were observed in the top portion of the CSA (toward the soil surface) for the roots belonging to all four quadrants. The bottom portion of the CSA showed the highest values for the roots belonging to the downslope and windward quadrants. Cumulative values of CSA of all the traces of the lateral roots belonging to the same quadrant also showed the highest values in the top portion with respect to the bottom portion. When cumulative CSA was measured in the four quadrants, the highest values were obtained for the roots belonging to the downslope and windward quadrants. In particular, the cumulative CSA measured for the roots belonging to the downslope quadrant showed a significantly higher value than those in the upslope quadrant and the CSA values measured for the roots belonging to the windward quadrant were significantly higher than those in the leeward quadrant.

**Figure 5:**
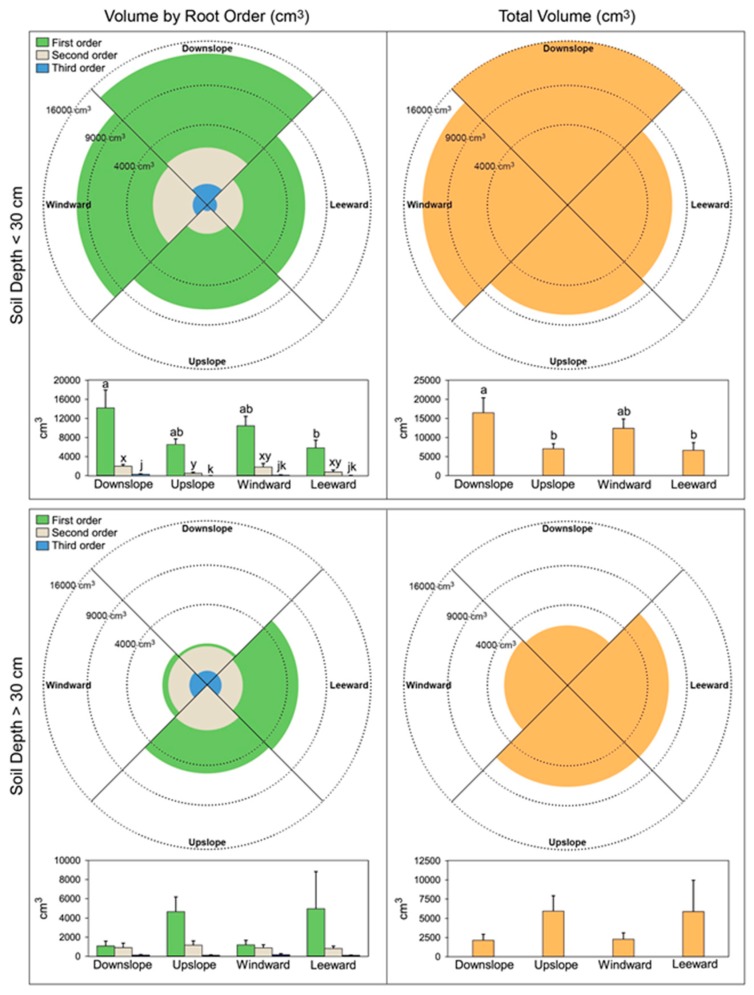
Volume of lateral roots present in the cage according to branching order (left column), pulled together (right column), and at different depths (<30 cm upper panels, >30 cm lower panels) in each quadrant. Letters within each order, quadrant, and soil depth combination indicate significant differences (*p* < 0.05); absence of letters reflects that no significant difference was detected. Vertical boxes represent approximately 50% of the observations and lines extending from each box are the upper and lower 25% of the distribution. Within each box, the solid horizontal line is the median value and the dotted line is the mean.

Regarding the root volume, the first-order lateral roots presented a higher volume in the downslope and windward quadrants with a significantly lower value in the leeward quadrant (Figure 5). Also, the volume presented by the second-order lateral roots showed a higher value in the downslope and windward quadrant, although differences were scarcely significant. Regarding the third-order lateral roots, the values observed were too low to be of any importance. At soil depth > 30 cm we found the opposite situation for the first-order lateral roots, with higher values found in both upslope and leeward quadrants. The value of the volumes of the second- and third-order lateral roots was very low. The pattern of volume values shown by the different branching order at both depths was more clearly visible when all lateral roots were put together (Figure 5).

### 3.2. Variation of the Eccentricity of the Annual Rings

**Figure 6:**
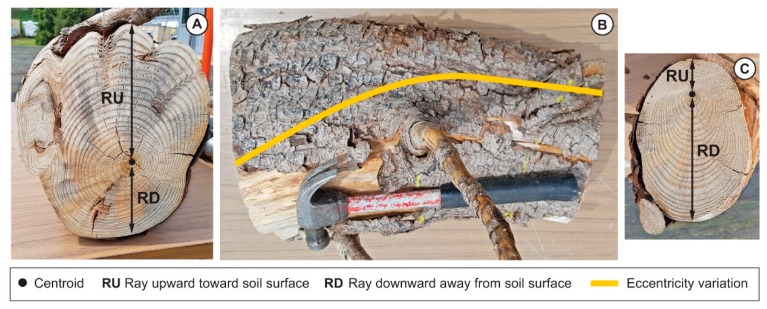
Variation in annual ring eccentricity in the shallow root within the cage. Panel A shows the cross-sectional area (CSA) at the branching point (**A**). Panel B shows the tapering of the lateral root axis from the branching point to the cage edge (**B**). The yellow line connects the two centroids of the CSA. Panel C shows the annual ring eccentricity at the cage edge (**C**).

We observed annual ring eccentricity in the traces left on the taproot after the lateral roots were removed, and also at the cuts on the lateral roots at the edge of the cage. The direction of the eccentricity at either end of the lateral roots was, however, completely different (panel A and C in Figure 6). In particular, the eccentricity at the taproot branching point showed an upward (toward the soil surface) direction (panel A in Figure 6), whereas at cage edge the eccentricity was directed downward, away from the soil surface (panel C in Figure 6). These results indicated that the change in annual ring eccentricity occurred within the length of the lateral root, coinciding with the distance from the branching point to the cage edge, and was visible as an upward transition of the centroid (panel B in Figure 6). The eccentricity of annual rings was accompanied by a different amount of early wood and latewood (Figure 2). In the portion of the annual rings showing the maximum eccentricity, the measurement of the thickness of early wood and latewood under the stereomicroscope showed that the ratio between the two wood types was always higher than 1 (data not shown). 

For three randomly selected roots, we cut four slices (2 cm thick) of wood perpendicular to the root axis. The first slice was at a distance of 2 cm from the taproot branching point, the second was 2 cm from the edge of the cage, and two more slices were cut, respectively, at a position one-third (named intermediate 1) and two-thirds (named intermediate 2) of the length measured between the branching point and the edge of the cage. When we measured the RU and RD length in the four wood slices (branching point, intermediate 1, intermediate 2, edge of the cage) of each root sample, we observed that the value of RU decreased whereas the value of RD increased as we proceeded from the branching point to the edge of the cage (Table 1). The sum obtained by adding RU to RD for each wood slice indicated that the roots underwent a considerable tapering in the distance, occurring between the branching point and the edge of the cage.

### 3.3. Modelling Mechanical Forces

**Figure 7:**
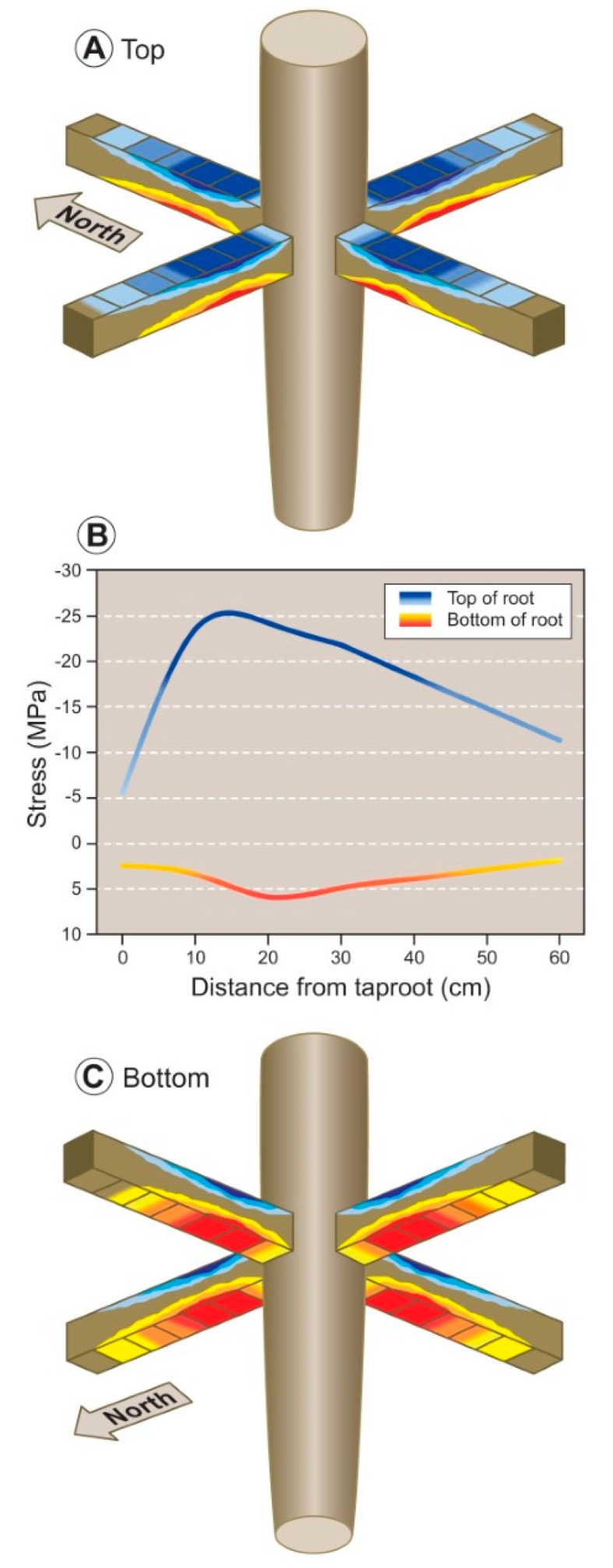
Model representing the distribution of mechanical forces along the top and bottom portion of four theoretical lateral roots extending in the four cardinal directions. Upper panel (**A**) shows the distribution of compression forces. The highest intensity of the blue color represents where the maximum compression force is present. The lower panel (**C**) shows the distribution of tension forces. The highest intensity of the red color represents where the maximum tension force is present. The theoretical lateral root is divided into 10 cm long blocks, extending from the branching point to the cage boundary. The central rod represents the taproot. Intermediate panel (**B**) indicates the distribution of theoretical compression and tension mechanical forces (MPa) on the upper (top of the root) and lower (bottom of the root) side of the lateral root along the distance from the branching point (cm).

The model showed an even distribution across the four quadrants of the mechanical forces due to the self-loading on the lateral roots. In particular, compression forces (negative values in Table 2 and blue colors in Figure 7A) were acting on the top portion and tension forces (positive values in Table 2 and red colors in Figure 7C) on the bottom portion of the four theoretical lateral roots.

These mechanical forces were higher near the branching point and decreased moving toward the cage boundary. The theoretical values of compression- and tension-forces are highlighted in Figure 7B, with a peak in forces occurring at 10 cm in the top portion of the root and around 20 cm in the bottom portion of the root. In general, the tension forces acting on the bottom portion of the lateral roots (red–yellow line) were of lower magnitude, almost fivefold smaller, then compression forces (blue–light blue line) occurring in the top portion (Figure 7B).

When mechanical forces were analyzed in the four quadrants and along each theoretical lateral root (Table 2), data showed that, in the top portion of the lateral roots elongating in the downslope quadrant, the mean values of mechanical forces were significantly higher than upslope. When the mechanical forces acting on the lateral roots elongating in the windward were compared with those elongating in the leeward quadrants, the compression forces (blue colors) acting on the top portion of the lateral root did not differ. Instead, the tension forces (red colors) acting on the bottom portion of the lateral roots were significantly higher in the windward quadrant with respect to the leeward quadrant. 

## 4. Discussion

### 4.1. Root System Architecture and Root Cage

In the last two decades, we conducted investigations on both the fine and coarse root fraction of several species of angiosperms and gymnosperms [10,23,56,57,58,59,60,61,62]. In the present work, our objective is to elucidate the effect of mechanical forces on tree anchorage with a long-term goal of providing information useful to modelers constructing reliable models that predict the level of resistance to environmental stressors as provided by a particular 3D root system architecture. In *P. ponderosa,* we found that mechanical forces, due to in situ wind or the slope of the terrain, modify the 3D root system architecture [10]. Danjon et al. [27,63] suggested that roots included in the cage were the most important to ensure a firm anchorage of a tree to the soil. In our study, the greater volume of shallow roots in the cage and within the first 30 cm of taproot depth suggests that the loading forces are particularly intense in the first few centimeters of soil. Such mechanical forces acting on the root system can be smoothly (and rapidly) transferred into the ground [64] through the shallow lateral roots before any root is broken or pulled from the soil [16,65]. In particular, we observed that the lateral roots belonging to the first branching order present the highest percentage of the total volume of the entire root system in the downslope and windward quadrants of the 3D root system architecture. This suggests that the roots belonging to these quadrants are affected. Interestingly, we recently showed [10] that the shallow roots present in the first 30 cm of taproot depth are those that originated along with the taproot during the first phases of seedling establishment, indicating that adaptation to mechanical stress begins during the first phase of root system development.

The rapid taper we observed in the shallow first-order lateral roots within the limit of the cage concurs with Stockes and Mattheck [14]. The large volume near the taproot could increase root–soil contact, allowing the shallow lateral roots to rapidly dissipate the loading forces without danger of being bent or broken, given that the bending strength of a root is proportional to its diameter to the third power [14]. Moreover, species with plate root systems have relatively stronger wood farther along the lateral root (at a distance of 50–100 cm along the root) compared to roots from heart or tap root systems, and the root–wood strength decreased farther away from the tree after a strength maximum had been reached [14]. In cages with few shallow lateral roots, the stability seems, therefore, more dependent upon a long taproot that could be assisted by a number of sinkers, with the taproot behaving like a pole inserted in the soil and the shallow lateral roots serving as guy lines [55,66]. Our data on lateral volume within the cage show a shift in the position of maximum volume: in the first 30 cm of the taproot, lateral root volume is greatest in the downslope–windward quadrants, whereas volume is greatest in the upslope–leeward quadrant at depths exceeding 30 cm, suggesting the formation of a 3D root system architecture of a bi-lateral fan shape, as reported by Chiatante et al. [62] and Lombardi et al. [63].

### 4.2. Mechanical Forces and Annual Ring Eccentricity

We observed considerable levels of eccentricity in the annual rings of shallow roots, manifested in an external eccentricity lost rapidly within the limit of the cage, i.e., through considerable taper. While Bodoque et al. [67] attributed this phenomenon in *Pinus sylvestris* to the loss of soil cover that exposed the root to direct atmospheric contact, this was not the case in our study because all the roots we examined were well below the soil surface and eccentricity was observed upward and downward, in contrast to atmospheric-induced asymmetry that is only observed upward. Stokes and Mattheck [14] suggested that tree stability increases when biomass allocation is distributed with a greater wood density in areas of the root system where mechanical stresses are stronger, with the consequent induction of eccentricity between the upper or lower sides of the root. These authors and others [68,69,70] suggested that the consequent formation of eccentrically shaped roots under mechanical stress improves tree stability, as these roots resist imposed bending stresses more efficiently than roots, with a more even distribution of secondary thickening around their circumference. Therefore, the eccentricity in root shape we observed in our shallow lateral roots could be regarded as a response to mechanical forces. Based on the research of Mattheck and Breloer [71] for roots of other conifers, we therefore suggest that reaction wood in *P. ponderosa* forms in response to mechanical forces and may be visible in the eccentricity of the annual rings and root shape.

Although Timell [35] suggests that annual ring eccentricity in stems and branches is usually associated with compression wood development, this is not always the case [35,72,73]. Compression wood has been classified or described using wood density, the qualitative and quantitative anatomical features, based on the chemical and physical organization of tracheid cell walls, wood color, and the ratio of late-to-early wood [34,35,74]. In our roots, we observed a darker color in an eccentric cross-section, similar to the results of Kim et al. for stems [75], and always in the top portion of the root section (radius toward the soil surface). However, our observation that the top portion of the root section in *P. ponderosa* (characterized by eccentricity in Figure 2B) was only slightly darker than that found in the opposite portion (opposite or normal wood in Figure 2B), combined with the observation that compression wood color varies across conifer species [34,35], leads us to concur with Bräuning et al. [53] that color alone cannot be used as a unique element for compression wood classification.

We noted low rates of taper within our sinker lateral roots, suggesting that they are under the influence of mechanical forces dissipated to the soil without any preferential direction (i.e., equally around the root girth), similar to the results noted for horizontal conifer stems artificially exposed to uniform mechanical forces [35]. Furthermore, a change in the direction of the annual ring eccentricity in our lateral roots from their branching points at the taproot to the extreme edge of the cage was also observed. As a close contact exists between the root surface and the soil, we believe this change in eccentricity is not caused by the slow torsional movement of the root, especially if one considers that this root segment is connected on one side to the taproot, and on the other side to the rest of the root system that extends for a considerable distance. Furthermore, this change in eccentricity direction cannot be interpreted as “spiral compression wood” that forms in rotating stems as described by Telewski [76] because the one-time change in eccentricity in our roots is 180° (i.e., from upward to downward direction). Therefore, we suggest that the change of eccentricity direction within the cage represents a response to a change in the mechanical forces acting on the root system (e.g., stem sway [38]). 

A hypothesis is that one force is dominant on the initial portion of the root axis from the branching point to the first intermediate point. In this case, the response to the mechanical force could be the induction of an eccentricity of annual rings in the top portion (upward direction) of the root cross-section. This force weakens with distance and near the second intermediate point is dominated by a second force that induces the formation of annual ring eccentricity in the bottom portion, with a downward direction. According to this hypothesis, the annual ring eccentricity, for the first centimeters after the branching point and in the top portion of the root, could assume the function of pushing the root into the soil to avoid exiting from the soil [65], whereas at a further distance (i.e., cage edge), and in the bottom portion, could assume the function of imposing on the root a continuation of horizontal growth.

### 4.3. Modeling of Mechanical Forces within the Root Cage 

Although the exact nature of these two forces remains unclear, modeling of the forces affecting our root systems shows a design that is coherent with our direct measurement. In particular, our model shows the occurrence of compression and tension forces, respectively, in the top and bottom portion of the root as results of the load due to self-loading (i.e., the weight of stem, branches, and leaves). Force distribution is similar in all quadrants, and this suggests that self-loading might be considered as the dominant source of mechanical force acting on the root systems independently from their topological orientation. These findings concur with the occurrence of annual ring eccentricity in the top portion of the root section (toward the soil surface) for all the shallow roots present in the four quadrants. Furthermore, our model suggests that the magnitude of negative and positive forces is greater closer to the branching point and dissipates as the root grows away from the branching point (i.e., approaching the cage edge), as one should expect, because the self-loading influence is decreasing. This dissipation of mechanical forces at an increasing distance from the branching point also explains our observed reduction in annual ring eccentricity. 

Low [77] suggested that compression wood in conifer stems forms in response to wind and slope, and our lateral root trait data appears to confirm this for roots. Despite compression forces acting within all four quadrants, their intensity is much higher in the downslope quadrant with respect to the upslope. Similarly, tension forces act on all quadrants too, but they are significantly higher in the windward quadrant with respect to leeward. The difference in the downslope–upslope comparison might be explained by assuming that, besides self-loading, another additional compression force is active only on the downslope quadrant, likely derived from the slope. In the case of windward–leeward comparison, the difference could be explained by assuming that the additional force is the wind, which increases the tension forces only in the windward quadrant. Low [77] further suggests that when wind and slope forces are acting together on the same tree, the stem response to wind dominates the response to slope, whereas our results suggest an opposite reaction in roots: the response to slope exceeds that of wind. 

## 5. Conclusions

For 32 year old *Pinus ponderosa* trees growing on slope conditions with a seasonally dominant wind, we analyzed their root cages in four quadrants (upslope, downslope, windward, and leeward) and used these data to explore the nature and intensity of mechanical forces acting on the root system through a theoretical model. Our intention is that our model can be integrated with other published models (reviewed by Moulia and Foirnier [45]) to achieve an improved understanding of how root architecture changes to increase root–soil anchorage. 

Our cross-section analysis, measured proximal to the branching point, showed that, independently of the quadrant analyzed, roots developed eccentrically with a higher vertical radius and cross-sectional area in the top portion of the root section, closer to the soil surface. However, this eccentricity inverted along the root axis toward the cage edge. Further, this eccentricity and overall root volume was higher in the downslope and windward quadrants with respect to their counterparts. The asymmetrical distribution of root volume was lower and opposite at the greater soil depth, showing classical fan-shape architecture. 

Along the root axis, our theoretical model showed that compression mechanical forces were acting on the top portion of the root whereas tension occurred in the bottom. While these forces were uniform around the modeled root system, the highest values for compression and tension were found downslope and windward, respectively. Also, forces were highest near the branching point and dissipated along the root axis. These findings suggest that mechanical forces might be responsible for the eccentrically wood production in the root section, and for the asymmetrical volume distribution around the root. 

We conclude that the self-loading of the above ground biomass acts as the main source of mechanical force triggering wood eccentricity in roots emanating from the taproot. The highest values of compression forces in the downslope quadrant may explain why the eccentricity is the highest. Also, the highest value of tension forces in the windward root might be equally responsible for the higher eccentricity by increasing the inhibition of cambium activity in the lower part of the root. Finally, the dissipation of forces along the root axis is in accordance with the change in direction of the eccentricity toward the cage edge. Thus, in addition to the self-loading intensity, the forces caused by wind and slopes are further superimposed, enhancing the wood eccentricity of roots growing in those quadrants. An interaction of compression (stimulation) and tension (inhibition) forces drives the asymmetrical cambial activity.

Our work highlights how, in response to mechanical forces originated from self-loading, slope, and wind, the root systems of *Pinus ponderosa* adopt a complex mechanism that includes an increase in root section eccentricity in combination with root volume asymmetry. These modifications progressively diminish along the root axis as mechanical forces dissipate.

## Figures and Tables

**Table 1:** A schematic of the eccentricity of three randomly selected roots and the actual radius lengths (cm). RD, radius length moving from the centroid (black dot in Figure 2) downward, away from the soil surface. RU, radius length moving from the centroid upward toward the soil surface. For each root, moving left to right is the branching point on the taproot to the cage boundary. The four dots from left to right represent, respectively, the position of the centroid: first dot on the left at the branching point; second dot from the left at 1/3 of the root length (intermediate 1), third dot from left at 2/3 of the root length (intermediate 2), fourth dot from the left at the edge boundary.

	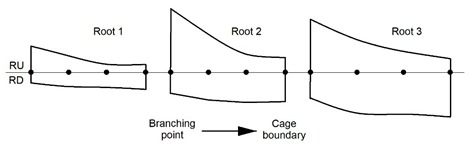					
**Location**	Root 1		Root 2		Root 3	
	RD	RU	RD	RU	RD	RU
Branching point	3.8	9.4	7.5	22.8	9.2	19.0
Intermediate 1	5.1	5.9	9.9	13.6	14.6	11.7
Intermediate 2	5.4	2.9	10.8	6.5	15.0	7.5
Cage boundary	6.1	2.8	10.5	5.3	16.0	4.9

**Table 2:** Values of compression (blue colors in Figure 7) and tension forces (red colors in Figure 7) acting on the four theoretical lateral roots extending in the four different 3D quadrants. Mean ± standard deviation of the mechanical force (MPa) distribution obtained by the model along the lateral root axis (0–60 cm). The differences in force intensity values measured in the upper (compression forces—blue colors) and lower side (tension forces—red colors) of each lateral root extending in the four 3D quadrants (upslope, downslope, windward, and leeward) are presented. P values are indicated in italic, and in bold only when indicate significant differences (*p* < 0.05).

	Quadrant			
	Upslope	Downslope	Windward	Leeward
Top: Toward soil surface	−17.08 ± 6.92	−17.10 ± 6.92	−17.09 ± 6.92	−17.09 ± 6.92
		***>0.0001***	***>0.0001***	***>0.0001***
			***>0.0001***	***>0.0001***
				*0.0714*
Bottom: Away from soil surface	3.60 ± 1.41	3.60 ± 1.41	3.60 ± 1.42	3.60 ± 1.41
		*0.1655*	***0.0045***	***0.0005***
			***0.0001***	***0.0046***
				***0.0004***
